# Effect of Chlorine Salt Content on the Microstructural Development of C-S-H Gels and Ca(OH)_2_ at Different Curing Temperatures

**DOI:** 10.3390/ma17184460

**Published:** 2024-09-11

**Authors:** Wenjie Qi, Zhisheng Fang, Shiyi Zhang, Yingfang Fan, Surendra P. Shah, Junjie Zheng

**Affiliations:** 1 School of Civil Engineering and Geomatics, Shandong University of Technology, Zibo 255000, China; 2 Institute of Road and Bridge Engineering, Dalian Maritime University, Dalian 116026, China; 3 Department of Civil and Environmental Engineering, Northwestern University, Evanston, IL 60208, USA; 4 Department of Civil Engineering, University of Texas at Arlington, Arlington, TX 76010, USA; 5 School of Civil Engineering, Wuhan University, Wuhan 430000, China

**Keywords:** chloride content, 50 °C curing, C-S-H gel, CH, gray correlation analysis

## Abstract

Freshwater resources are scarce in coastal areas, and using seawater as mixing water can alleviate the scarcity of freshwater resources. However, the presence of chloride ions in seawater affects the generation of hydration products and the durability of concrete structures. In order to investigate the effect of hydrated calcium silicate (C-S-H) gel and calcium hydroxide (CH) generation in seawater-mixed cement pastes under 50 °C curing, their microscopic morphology was investigated using differential scanning calorimetry analysis, X-ray diffraction (XRD), and scanning electron microscopy (SEM). The relationship between the amount of C-S-H gel and CH production and the amount of chloride ion dosing, fly ash dosing, and the age of curing were investigated. The degree of influence between hydration products and influencing factors was analyzed using the grey correlation analysis. It was shown that 50 °C curing promoted the hydration reaction and generated more hydration products compared with ASTM standard. The content of C-S-H gel and CH increased with chloride dosage. The content of C-S-H gel increased by 13.5% under 50 °C curing compared with the control group at a chloride dosage of 1.3%. Fly ash is rich in active SiO_2_ and AI_2_O_3_, and other components, which can react with Ca(OH)_2_ generated by cement hydration and then generate C-S-H gel. With the increase of fly ash, the content of C-S-H gel also increases, but the CH content decreases. When 25% of fly ash was doped under 50 °C curing, the C-S-H gel content increased by 5.02% compared to the control group. The CH content decreased by 31.8% compared to the control group. With the growth of the maintenance age, the hydration reaction continues, the generation of C-S-H gel and CH will continue to increase, and their microstructures will become denser. C-S-H gel and CH content increased the most by raising the curing temperature at 7 days of curing, increasing by 10.11% and 22.62%, respectively. C-S-H gel and CH content had the highest gray relation with fly ash dosing. Chloride dosage and age of maintenance had the highest correlation with CH content at room temperature maintenance of 0.788 and 0.753, respectively.

## 1. Introduction

With the development of China’s marine industry and the strategy of moving towards the deep blue, infrastructure construction on wharves, ports, and islands in coastal areas is developing rapidly. There is a lack of freshwater resources in coastal areas [1].

Transporting fresh water from the mainland to coastal areas requires a lot of manpower, material resources, and time, and increases costs. In addition, there are a large number of seawater resources in the coastal areas of China, and using seawater for engineering construction will produce significant economic benefits [2]. The use of seawater instead of fresh water for use as mixing water can reduce the use of fresh water, and coastal area infrastructure construction using seawater can be done with both local materials. It can also effectively reduce the cost and, at the same time, promote the sustainable development of resources. As seawater is rich in dissolved salts, it will bring in chloride ions when used to formulate concrete, which will affect the hydration process of cement and product generation, create or change the concrete structure, and affect the performance of the concrete [3]. Therefore, it is necessary to study the effect of seawater mixing on cement hydration products and the effect of different curing temperatures and additives.

In recent years, seawater-mixed concrete has become a hot research topic for some scholars, including the reasons for the accelerated hydration of seawater [4,5,6]. For example, the chemical reactions of chloride ions in concrete (for example, the formation of Friedel’s salt or chloride and its effects on C-S-H generation) [6,7]; changes in the C-S-H gel (for example, its composition, morphology, micro-mechanical properties, chemical composition, chemical properties, and chemical properties) [8,9]; and the effect of other ions in seawater (SO_4_^2−^, CO_3_^2−^, Na^+^, Ca^2+^, mg^2+^) on hydration products and chloride binding [4,7,10], etc. In the study of the effect of seawater mixing on the hydration kinetics of concrete, it was found that the incorporation of seawater increased the hydration of cement by 4.67% and increased the average chain length of the C-S-H gel, thus increasing the degree of polymerization of hydration products [1].

C-S-H gel plays a crucial role in cementitious materials by having an important influence on the durability, mechanical properties, and cohesive behavior of the materials [11] and by controlling the main chemical and physical properties of hardened cementitious materials [12,13]. Numerous researchers and scholars have concluded that C-S-H gels dominate in the hydration products of ordinary silicate cement by the physisorption of chloride ions [14,15]. Cation chemistry in chloride-containing solutions can also alter chloride binding [16,17,18]. The chloride binding capacity of gelling materials exposed to CaCl_2_ and MgCl_2_ solutions is stronger than that of NaCl solutions [16,19]. This increase in chloride binding capacity mainly originates from the increased physical adsorption of chloride ions by the C-S-H gels [20,21]. Under some special conditions, such as high initial chloride concentrations and relatively low temperatures, the electrostatic attraction of CH, calomel (AFt), and chloride-rich AFm phases (Cl^−^-AFm) is also small, resulting in less chloride binding [22,23]. Numerous studies have reported that adsorbed calcium ions control chloride binding associated with C-S-H and are closely related to solution pH [20,23].

Otsuki [24] concluded that the physisorption of chloride ions is highly correlated with the calcium/silica (Ca/Si) ratio of the C-S-H gel. A higher Ca/Si ratio effectively accelerates the physisorption of chloride ions. Honglei Chang [25] showed that the effect of mineral admixtures on the C-S-H chloride binding capacity is the same as that of cement paste. The effect of fly ash on chloride binding capacity depends on the concentration of the NaCl solution. CH shows a laminated shape under standard curing, a hexagonal plate shape under high temperature curing, and a lamellar structure during high pressure steam curing [26]. Alhozaimy [27] found that the CH content in the matrix after high temperature curing was significantly lower than that after standard curing. In addition, high temperature curing can accelerate the hydration process between mineral admixtures and CH to produce more C-S-H gels, improving strength and durability [28]. The curing temperature has an important effect on the hydration reaction of silicate cement, and it is not an exception for cement containing volcanic ash blends. Increasing the maintenance temperature can promote the volcanic ash reaction of fly ash, a volcanic ash blend [29], and change the microstructure of the hardened cement paste [30].

The above studies still lack a clear understanding of whether the curing temperature significantly affects the participation of chloride ions in the hydration reaction and the degree of volcanic ash reaction of fly ash. In order to solve the problem of freshwater scarcity in coastal areas, to clarify the effect of seawater on the hydration products of cement, and to analyze the effects of mineral admixtures and curing temperatures, this paper investigates the hydration process of cement paste mixed with chloride ions and fly ash at temperatures of 20 °C and 50 °C. Aiming to solve the problem of replacing fresh water with seawater in concrete mixing in practical projects, experts at home and abroad have conducted many experimental studies. Most of the research is on mechanical properties, and there is less research on the hydration products of seawater concrete. In this experiment, a scanning electron microscopy (SEM) scanning electron microscope was used to observe the microstructure of concrete specimens and X-ray diffraction (XRD) and differential scanning calorimetry (DSC-TG) analysis of seawater mixing on hydration products. The effects of chloride ion concentration, fly ash admixture, and curing age on the hydration products of chloride-containing cementitious materials were analyzed from the internal micro level. The degree of influence between the hydration products of chloride-containing cementitious materials and the chlorine ion dosage, fly ash dosage, and curing age at different curing temperatures was also quantified by the gray correlation analysis degree method.

## 2. Materials and Methods

### 2.1. Test Materials

**Figure 1:**
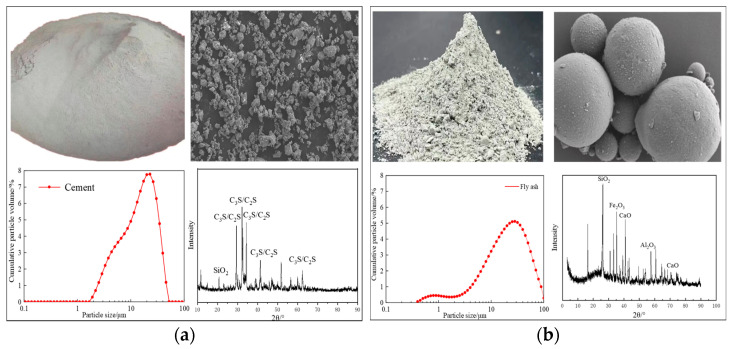
Microscopic properties of experimental materials. (**a**) Cement (**b**) Fly ash.

Shandong Dongyue brand PO42.5 grade ordinary Portland cement (OPC) was used in this experiment, and the properties of the cement are shown in Table 1 and Figure 1a. The fly ash is F-grade fly ash; its properties are shown in Table 1 and Figure 1b. The water reducing agent was polycarboxylic acid high efficiency water reducing agent, and the mixing water was NaCl solution.

### 2.2. Specimen Preparation and Mixing Ratio

**Figure 2:**
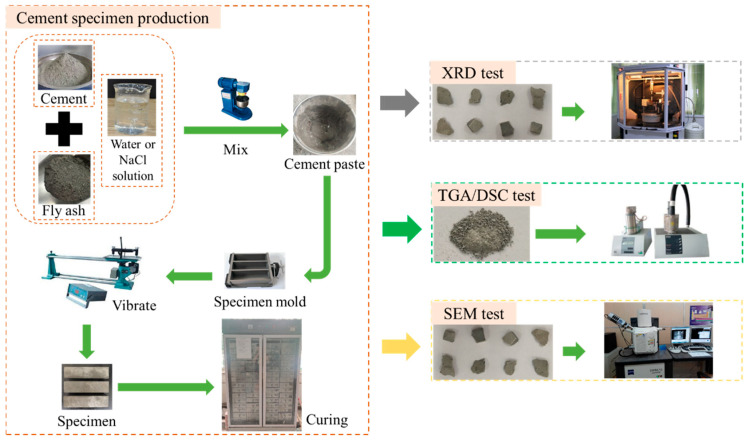
Fabrication flow chart.

The experiments prepared cement net mortar specimens by adding cement and fly ash to a programmable mixer and mixing thoroughly. NaCl particles were added proportionally to the water and dissolved homogeneously using the mixer. This resulted in a simulated seawater solution. Slow mixing for 2 min and fast mixing for 2 min were used to ensure the materials were well mixed. Then, the mixed cement paste was poured into a 40 × 40 × 160 mm mold for molding. The specific process is shown in Figure 2. The specimens were demolded after 1 day and then placed into water bath curing boxes with different temperatures for curing according to ASTM standard [31], respectively. We carried out the curing for 3, 7, and 28 days, and the specimens were taken out at each time point for testing and analysis. The specimen fit ratios are shown in Table 2.

### 2.3. Test Methods and Procedures

#### 2.3.1. SEM

After the specimens at different curing temperatures were cured, the specimens were broken into flat sample pieces with an area of about 5 mm × 5 mm. The middle part was soaked in anhydrous ethanol to terminate hydration. The terminated hydrated samples were put into an oven for drying, and the oven temperature was set at 50 °C. After drying, the samples were sprayed with gold and subjected to SEM tests. The samples were observed using a Hitachis-4800 scanning electron microscope manufactured by Hitachi (Hitachi, Tokyo, Japan) with an operating voltage of 10.0 KV.

#### 2.3.2. XRD

The specimens with different curing methods were crushed and the middle portion was selected to be ground to a certain fineness. The sample powder was stacked on the sample stage according to the sample preparation requirements of the Panacor Sharp Shadow X-ray diffractometer (Malvern Panalytical, Shanghai, China). We ensured that the sample surface was flat and of uniform thickness to avoid overlapping of diffraction peaks or reduction of intensity due to too thick a sample. The scanning speed was set to 10°/min. We compared the XRD results obtained from the different curing methods to analyze how the curing conditions affected the crystal structure and properties of the material. This may involve the transformation of the crystalline phase, grain growth, and changes in the stress state.

#### 2.3.3. DSC-TG Analysis

DSC-TG analysis was performed on seawater-mixed cement paste powder samples. A TG-DSC STA 409 thermogravimetric analyzer was used to determine the content of hydration products (C-S-H gel, CH) in seawater-mixed cement pastes. In the test, samples were first taken from the core of the specimen, pulverized, and then terminally hydrated with anhydrous ethanol for 1 day. The prepared samples were then dried in a vacuum oven at 105 °C for 4 h and ground to a fineness of less than 150 μm. An amount of 30 mg of the sample was tested and heated from 25 °C to 1000 °C using a nitrogen atmosphere at 10 °C/min.

## 3. Analysis of Results

### 3.1. Effect on C-S-H Gel under Different Influencing Factors

#### 3.1.1. Effect of Chloride Ion on C-S-H Gel

**Figure 3:**
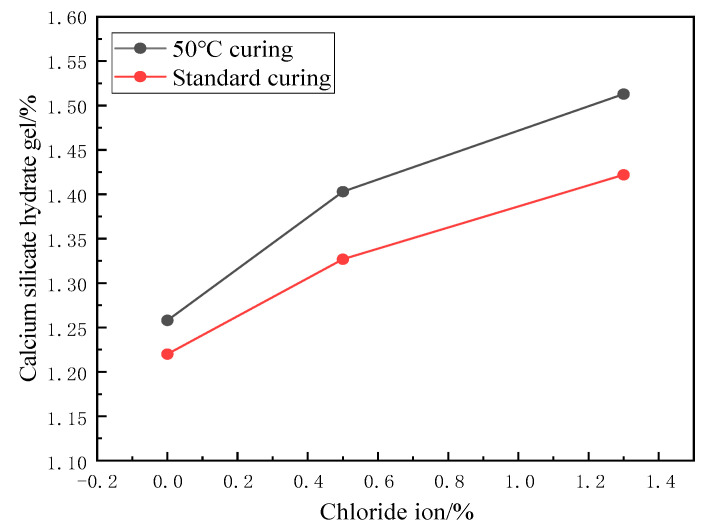
Effect of chloride ion concentration on the relative content of C-S-H gel.

**Figure 4:**
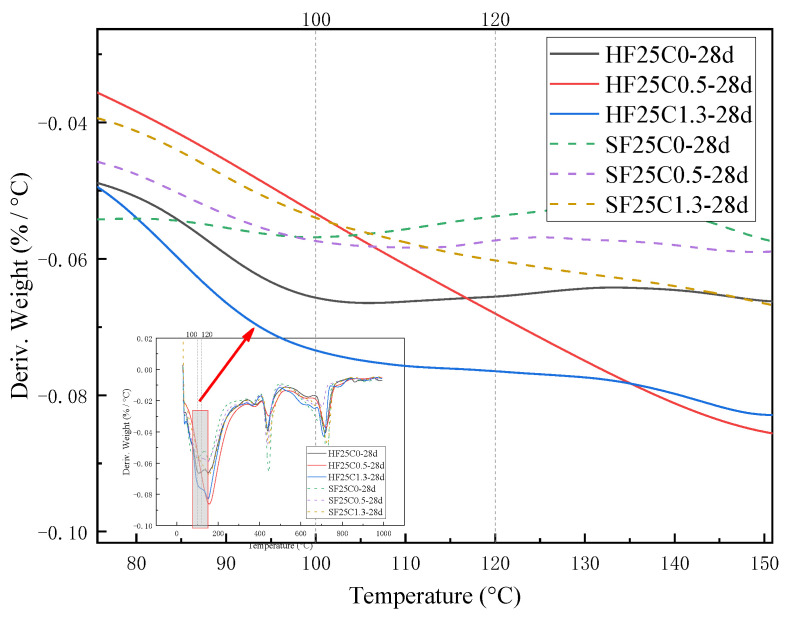
Effect of chloride ion concentration on the heat-absorption decomposition peak of C-S-H gels.

Figure 3 and Figure 4 show the effect of chloride ion concentration on C-S-H gel content at different temperatures. As seen in Figure 4, three obvious weight loss processes in the samples were observed during the warming process from 30 to 1000 °C. The weight loss at 100~120 °C is due to the evaporation of adsorbed water in the C-S-H gel and the dehydration of gel water [32]. The C-S-H gel was then thermally decomposed into wollastonite (CaSiO_3_).

As can be seen from Figure 3 and Figure 4, the content of the C-S-H gel of cement paste after chloride ion doping under 50 °C curing is more than that of the control group. With the increase of chloride ion doping, the C-S-H gel content also gradually increased; the C-S-H gel content in the 50 °C curing of chloride ion doping increased by 1.3%, and the control group increased by 16.85%. At 1.3% chloride dosage under standard curing, the C-S-H gel content increased by 14.2% compared to the control group. This indicates that the presence of chloride ions affects a certain hydration reaction of cement and promotes the formation of C-S-H gel. Sun [4] found that adding a single salt increases the concentration of calcium compounds in the solution, which helps to increase the formation of C-S-H gel at an early stage. Similar findings were also reported by Cai [33].

Under standard curing conditions, the rate of cement hydration reaction was slower, and the generation of C-S-H gel was slower. The C-S-H gel’s physical adsorption of chloride ions was lower compared to the 50 °C curing.

**Figure 5:**
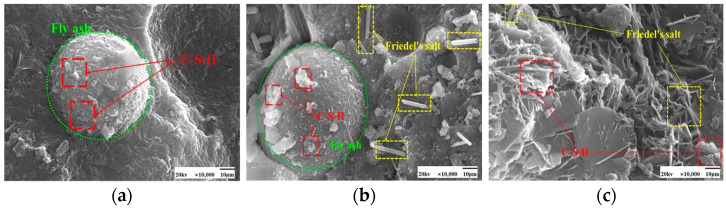
SEM images of a hardened slurry of cementitious materials with different chloride dosages for 50 °C curing. (**a**) 0% (**b**) 0.5% (**c**) 1.3%.

**Figure 6:**
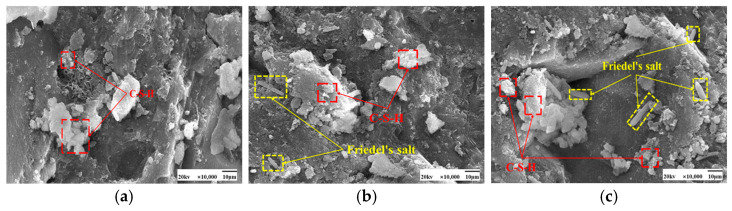
SEM images of a hardened slurry of cementitious materials with different chloride dosages in standard curing. (**a**) 0% (**b**) 0.5% (**c**) 1.3%.

SEM images of the effect of different chloride ion concentrations on the C-S-H gel of the hydration product are shown in Figure 5 and Figure 6. In both Figure 5a and Figure 6a, there is no doping of chloride ions, and also no generation of F salts. The admixture of chloride ions in Figure 5b,c and Figure 6b,c revealed the presence of Friedel’s salt. Friedel’s salt is the main product of the chemical combination of Cl^−^ with hydration products and consists of Cl^−^ with C_3_A, C_4_AF, or AFm interactions obtained as a lamellar crystal structure [34]. Cl^−^ enters the concrete as an internal admixture. Cl^−^ generates Friedel’s salt mainly through direct reaction with the aluminum phase [35]. Under 50 °C curing conditions, many amorphous C-S-H gels with a relatively compact overall structure and fewer pores were observed attached to the fly ash surface in these images. Compared with standard curing, more C-S-H gels were generated in the cement paste under 50 °C curing, and the distribution of these gels was more tightly arranged with better densification. This may be due to the action of chloride ions under 50 °C curing conditions. The presence of chloride ions may have promoted the formation of C-S-H gels in the cement paste.

#### 3.1.2. Effect of Fly Ash on C-S-H Gels

**Figure 7:**
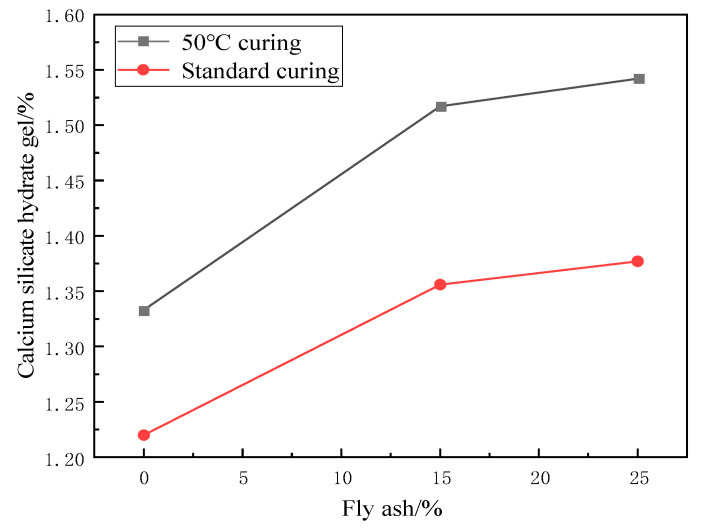
Effect of fly ash on the relative content of C-S-H gel.

**Figure 8:**
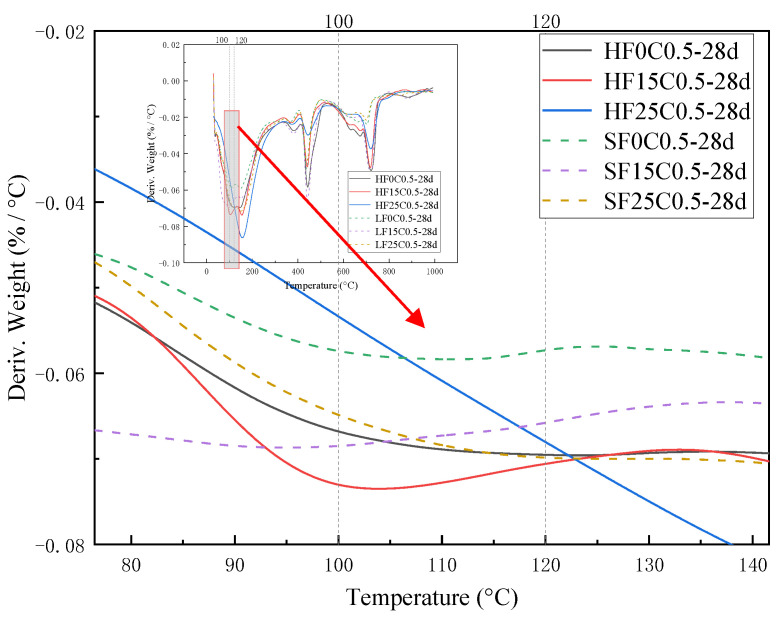
Effect of fly ash dosage on the heat-absorption decomposition peak of C-S-H gels.

As can be seen in Figure 7 and Figure 8, the same phenomenon was observed under 50 °C curing and standard curing conditions: the content of C-S-H gel increased with the increase of fly ash dosing. When the fly ash admixture was 25%, the content of C-S-H gel increased by 13.55% under 50 °C curing conditions and 11.4% under standard curing conditions compared to the control group. This is because fly ash is rich in active SiO_2_ and Al_2_O_3_, which can react with Ca(OH)_2_ generated by cement hydration to produce C-S-H gel. As a common mineral admixture, fly ash contains many fine particles and active ingredients, which can effectively participate in the cement hydration reaction. The reaction formulae are shown in (1)–(3).

(1) $$SiO_{2}+Ca{\left(OH\right)}_{2}+H_{2}O\to CaO\cdot SiO_{2}\cdot xH_{2}O$$

(2) $$Al_{2}O_{3}+Ca{\left(OH\right)}_{2}+H_{2}O\to CaO\cdot Al_{2}O_{3}\cdot xH_{2}O$$

(3) $$Al_{2}O_{3}+Ca{\left(OH\right)}_{2}+2SiO_{2}+3H_{2}O\to CaO\cdot Al_{2}O_{3}\cdot 2SiO_{2}\cdot 4H_{2}O$$

Under the 50 °C curing state, the hydration reaction in cement is rapid, releasing many hydrates. At this time, the active ingredients in the fly ash can react quickly with these hydrates to produce more C-S-H gels, thus increasing their content. In the standard maintenance state, the hydration reaction rate is slower but still occurs. The active ingredients in fly ash can still react slowly with Ca(OH)_2_ in cement to produce more C-S-H gels.

In summary, the content of C-S-H gel increased with the increase of fly ash dosage under 50 °C curing and standard curing conditions. This is because the active components in fly ash can react with Ca(OH)_2_ in the hydration reaction of cement to generate more C-S-H gels.

**Figure 9:**
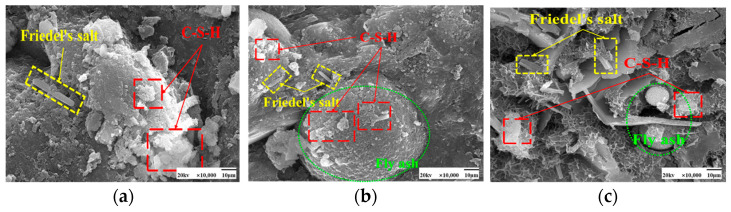
SEM images of a hardened slurry of cementitious materials with different fly ash dosages in 50 °C curing. (**a**) 0% (**b**) 15% (**c**) 25%.

**Figure 10:**
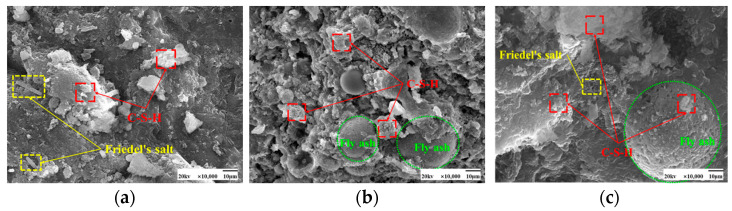
SEM images of a hardened slurry of cementitious materials with different fly ash dosages in standard curing. (**a**) 0% (**b**) 15% (**c**) 25%.

As seen in Figure 9 and Figure 10, the fly ash particles have begun to react with the cement during standard curing. The surface of the particles appeared as an etching phenomenon. However, there are still fly ash particles, the surface is still smooth, and nothing has yet appeared in the reaction phenomenon. At 50 °C curing, more C-S-H gel appeared on the surface of fly ash, and many hydration products appeared. Some larger pores are gradually filled, and the microstructure is much denser. Moreover, with the increase in the dosage of fly ash, there are also signs of volcanic ash reaction, forming a more dense and stable structure than the standard curing.

The activity of fly ash gradually increased with the increase of fly ash dosage in both standard and 50 °C curing. Chemical components in fly ash, such as silicates and aluminates, can react with water and calcium compounds in the cement to form C-S-H gels, which promotes the hydration reaction and produces more hydration products. These hydration products fill the larger pore structure within the cementitious materials and form smaller pores, enhancing the densification and impermeability of the cementitious materials [29].

#### 3.1.3. Effect of Maintenance Age on the Relative Content of C-S-H Gel

**Figure 11:**
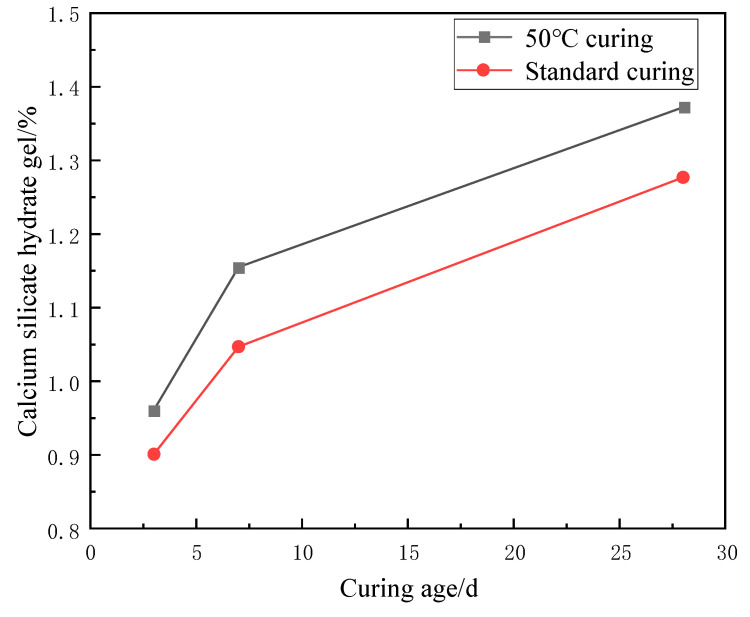
Effect of age of maintenance on the relative content of C-S-H gels.

**Figure 12:**
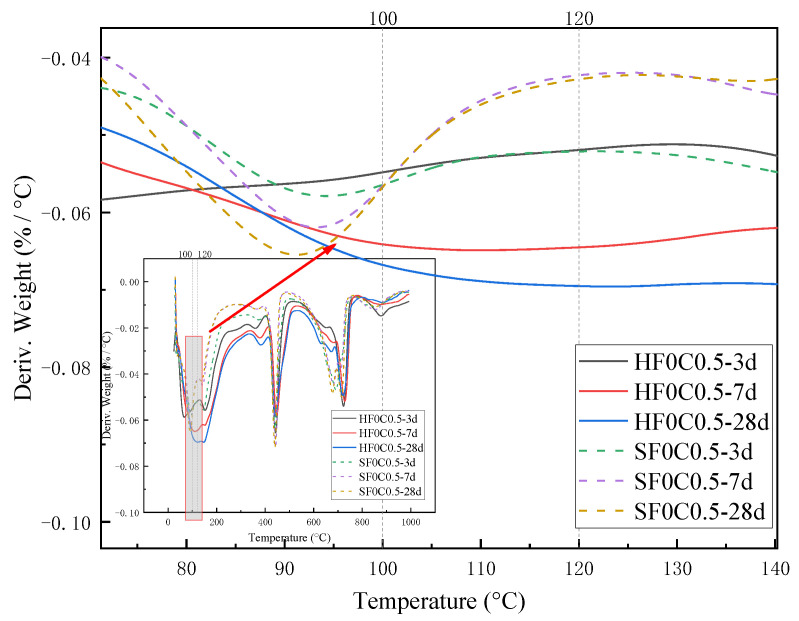
Effect of curing age on endothermic decomposition peak of C-S-H gel.

As can be seen from Figure 11 and Figure 12, under 50 °C curing, the increasing C-S-H gel generation was observed with the increase of the conditioning age. Under the same curing time, the 50 °C curing increased by 6.24%, 9.35%, and 6.92% at 3, 7, and 28 days, respectively, compared to the standard curing. This is because high temperature environment can promote the hydration reaction of cement and accelerate the formation process of C-S-H gel. With time, the hydration reaction continued, leading to a gradual increase in the generation of C-S-H gel. Similarly, the same trend was observed under standard curing conditions. Although the reaction rate was slower, increasing C-S-H gel generation could still be observed.

The effect of curing age on the amount of C-S-H gel generation is a gradual and cumulative process. With the increase of the curing time, the reaction products in the cement were gradually formed and accumulated, which led to a gradual increase in the amount of C-S-H gel generated. Therefore, prolonged 50 °C curing or standard curing can further increase the content of C-S-H gel and enhance the mechanical properties and durability of cementitious materials.

In summary, the trend of increasing C-S-H gel generation was observed with the increase in the age of curing, both under 50 °C curing and standard curing conditions. This is due to the continuation of the hydration reaction, which generates C-S-H gel.

**Figure 13:**
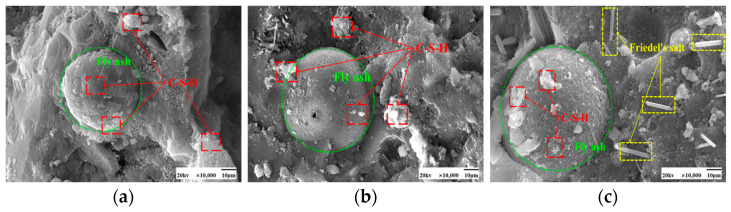
SEM images of C-S-H gels at different curing ages at 50 °C curing. (**a**) 3 days (**b**) 7 days (**c**) 28 days.

**Figure 14:**
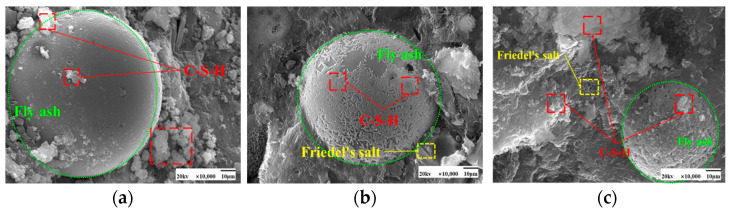
SEM images of C-S-H gels at different curing ages in standard curing. (**a**) 3 days (**b**) 7 days (**c**) 28 days.

Figure 13 and Figure 14 show the microscopic morphology of hardened slurries of cementitious materials at different curing ages. The same trend was observed for 50 °C curing and standard curing at different curing ages. Fly ash at the age of 3 days appeared to have fewer hydration products; fly ash particles in the aluminum-silica vitreous have begun to react with the hydration products of the cement particles on the surface of the etching phenomenon. However, there are still fly ash spherical particles whose surface is still smooth, and nothing has yet appeared in the reaction phenomenon; the volcanic ash reaction of fly ash has not been fully carried out. At 7 days, more C-S-H gel appeared on the surface of fly ash. Its microstructure had been much denser, and many hydration products had appeared in the system, and the pores were gradually filled. At 28 days, the surface of fly ash was wrapped entirely by the hydration products of cement and also showed signs of volcanic ash reaction, forming a more dense and stable structure than at 3 days and 7 days.

### 3.2. Analysis of Different Influencing Factors on CH Content

#### 3.2.1. Effect of Chloride Ion on CH Content

**Figure 15:**
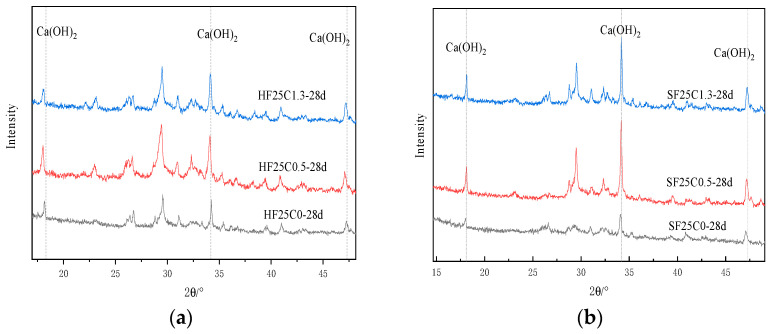
XRD analysis of the effect of chloride doping on CH generation: (**a**) 50 °C curing (**b**) Standard curing.

**Figure 16:**
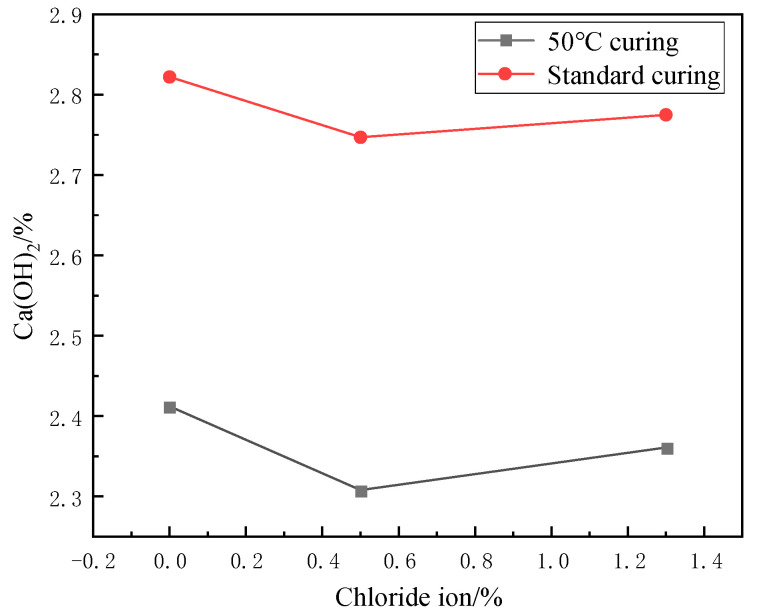
Effect of chloride ions on CH content.

**Figure 17:**
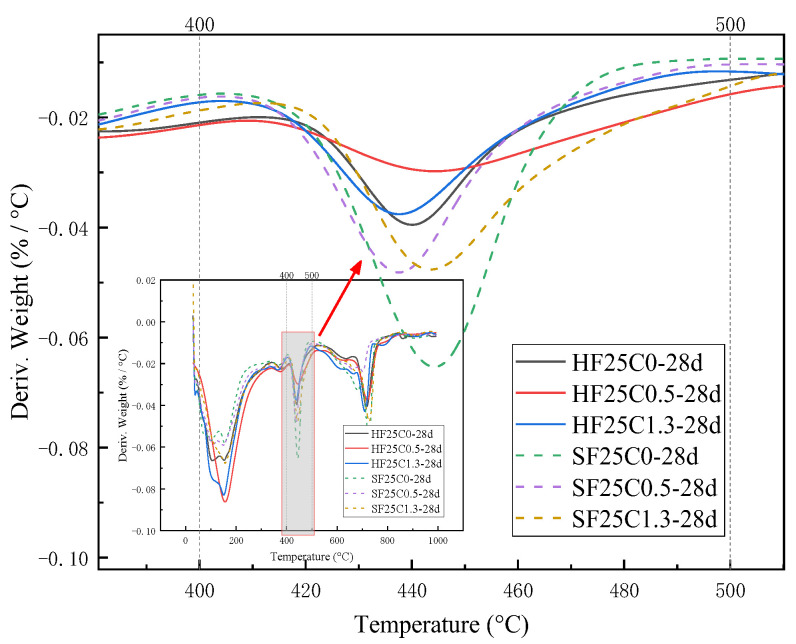
Effect of chloride ion concentration on the heat-absorption decomposition peak of CH.

**Figure 18:**
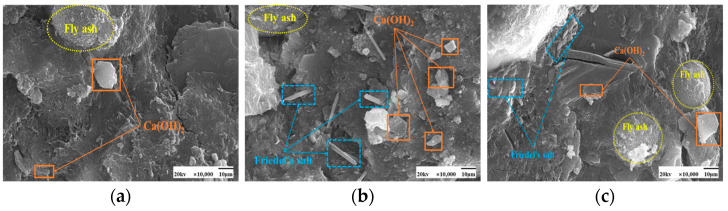
SEM images of a hardened slurry of cementitious materials with different chloride dosages in 50 °C curing. (**a**) 0% (**b**) 0.5% (**c**) 1.3%.

**Figure 19:**
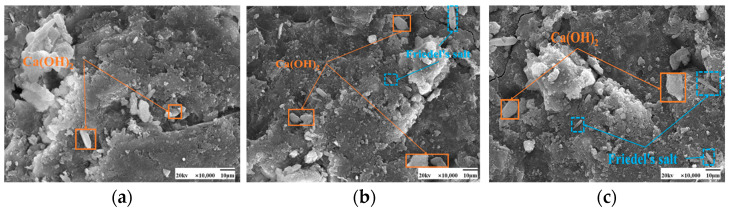
SEM images of hardened slurry of cementitious materials with different chloride dosages in standard curing. (**a**) 0% (**b**) 0.5% (**c**) 1.3%.

Figure 15a shows the XRD images under 50 °C curing conditioning. Figure 16 and Figure 17 show the changes in CH content at different curing temperatures. The study results show that after adding chloride ions, the diffraction peaks of CH were all increased compared to the control group. In particular, the most significant increase in the diffraction peak of CH was observed when chloride ions were doped at 0.5%. Traetteberg [36] investigated different types of cementitious net mortar specimens immersed in NaCl solution for 21 days using DTA-TG and XRD and found that CH immersed in NaCl solution binds some chloride ions. This indicates that as the concentration of chloride increases, CH consumes more chloride ions. Chloride ions in seawater mixes are involved in the early hydration reaction of cement as internal chloride ions. It first generates calcium chloride with CH (produced by the hydration reaction of tricalcium silicate). Then, calcium chloride reacts with tricalcium silicate to produce Friedel’s salt, which can be confirmed in Figure 18b,c and Figure 19b,c after chlorine ions are doped to produce Friedel’s salt. When the content of tricalcium silicate decreases at the later stage of the reaction, monochloride calcium chloroaluminate is formed. On the other hand, chloride ions can increase the solubility of cement clinker in the silicate phase, thus accelerating cement hydration [36]; the main reaction equations are shown in (4)–(7):

(4) $$2(C_{2}S)+6H_{2}O\to C_{3}S_{2}H+3Ca{\left(OH\right)}_{2}$$

(5) $$Ca{\left(OH\right)}_{2}+2Cl^{-}\to CaCl_{2}+2OH^{-}$$

(6) $$C_{3}A+CaCl_{2}+10H_{2}O\to 3CaO\cdot Al_{2}O_{3}\cdot 3CaCl_{2}+32H_{2}O$$

(7) $$C_{3}A+CaCl_{2}+10H_{2}O\to 3CaO\cdot Al_{2}O_{3}\cdot CaCl_{2}\cdot 32H_{2}O$$

The diffraction peaks of CH under 50 °C curing conditioning were all reduced to different degrees with the same doping of chloride ions. This indicates that the increase in temperature promotes the reaction of CH and consumes part of the CH, leading to a decrease in content.

The weight loss from 400 °C to 500 °C is caused by the dehydration of the CH, and the amount lost is the amount of water dissipated [37]. As can be seen from Figure 16 and Figure 17, the trend of the CH content was observed to decrease first and then slightly increase under 50 °C curing and standard curing conditions. The CH content reached the minimum value when the chloride ion concentration was 0.5%. The CH content was lower than standard conditioning at 50 °C curing. Increasing the temperature accelerated the above reaction and consumed the CH.

More plate-like CH appeared under 50 °C curing and standard curing. From Figure 18 and Figure 19, it can be seen that there is no significant change in the microscopic morphology of the CH after the incorporation of chloride ions. Moreover, the generation of Friedel’s salt was found in the figure after the addition of chloride ions.

#### 3.2.2. Effect of Fly Ash on CH Content

CH in cement paste is generally mainly generated by the hydration of calcium silicate. A secondary reaction with active fly ash will consume it. Therefore, the change in CH content is the combined effect of the generation of CH by the hydration of cement and its consumption by reaction with active fly ash. When the consumption of CH is greater than the production, the CH content in the slurry will decrease. Therefore, the change in CH content can be used to observe the cement hydration process and the degree of mixed material activity.

**Figure 20:**
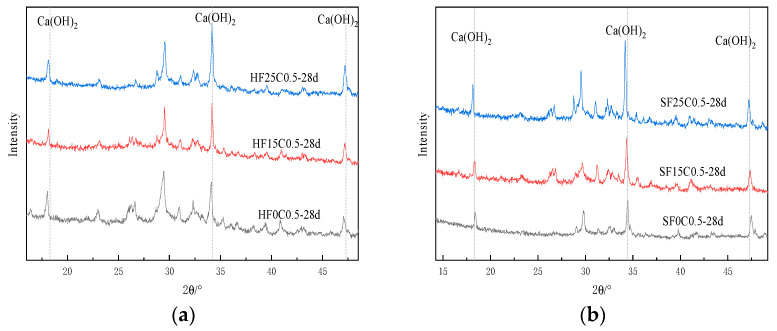
XRD plot of the effect of fly ash dosage on CH content. (**a**) 50 °C curing (**b**) Standard curing.

Figure 20 shows the XRD image of the effect of different fly ash dosages on CH content. The image shows that the diffraction peaks corresponding to the CH crystal structure under 50 °C curing and standard curing gradually decrease with the increase of the fly ash dosage. This indicates that the CH content is gradually decreasing, per the conclusion mentioned above.

In concrete, CH is an important product during the hydration reaction of cement. Under 50 °C curing and standard curing, the effect of an increase in fly ash admixture on CH content is the same. As the fly ash dosage increased, the silica reacted more fully with the CH in the cement, thus reducing the CH content.

**Figure 21:**
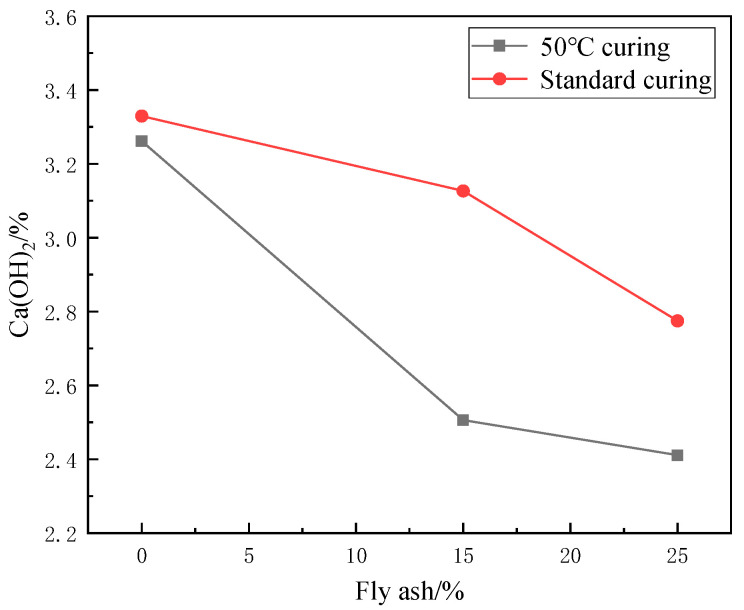
Effect of fly ash admixture on CH content.

**Figure 22:**
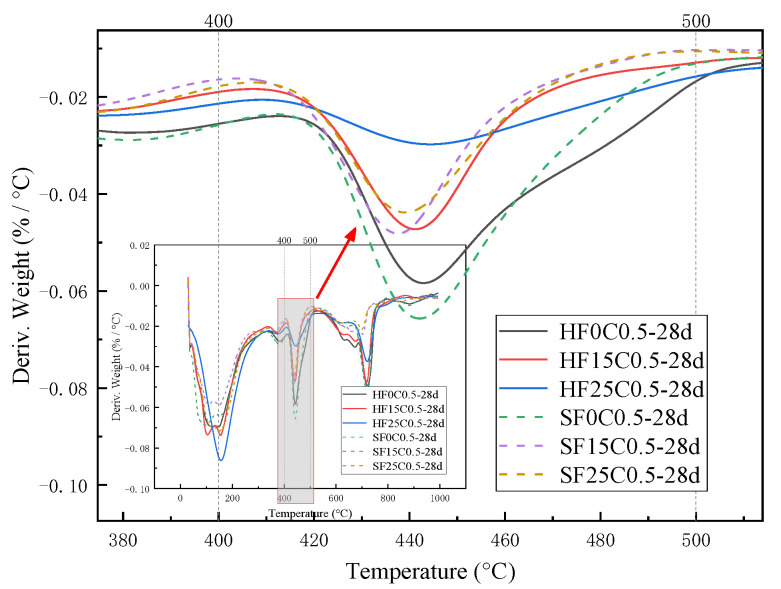
Effect of fly ash content on endothermic decomposition peak of CH.

As can be seen from Figure 21 and Figure 22, the CH content in the hardened cement paste decreases gradually with the increase of fly ash admixture. When the dosage of fly ash reaches 25%, the CH content decreases by 34.18% under 50 °C curing compared to the control group. The CH content decreased by 20.1% under standard curing compared to the control group. This is because the most abundant chemical in fly ash is silica, which has good volcanic ash activity. In concrete, silica reacts with CH and releases heat, promoting the cement’s hydration. This process effectively refines the grains of CH. It generates a C-S-H gel with strong cementation and a large specific surface area. Generating the C-S-H gel increases the strength of the cement and reduces the CH content. In addition, the generation of the C-S-H gel resulted in an improved structure of the transition zone in the concrete, which improved the bond between the cement paste and the aggregate. This further improved the strength performance of the concrete.

In summary, increased fly ash in concrete decreases the CH content in concrete. This is due to the high silica content in fly ash, which reacts with CH and promotes the hydration of the cement. This produces a C-S-H gel that enhances the cement’s strength and improves the concrete’s structure, which further improves the strength of the concrete.

**Figure 23:**
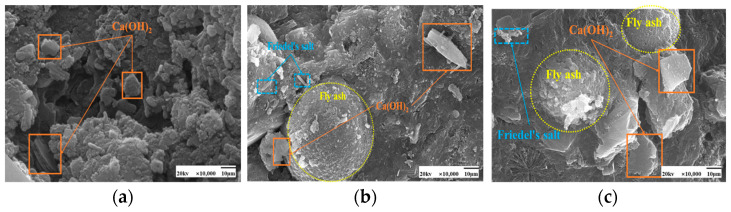
SEM images of a hardened slurry of cementitious materials with different fly ash dosages at 50 °C curing. (**a**) 0% (**b**) 15% (**c**) 25%.

**Figure 24:**
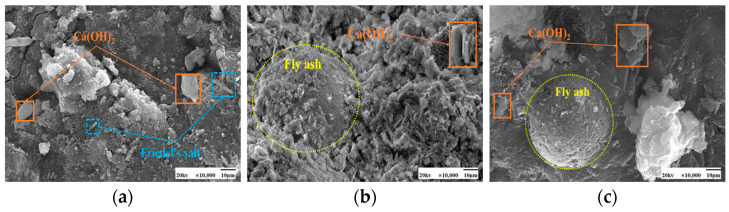
SEM images of CH with different fly ash admixtures in standard curing. (**a**) 0% (**b**) 15% (**c**) 25%.

SEM images of the effect of different fly ash dosages on CH are shown in Figure 23 and Figure 24. Etching was observed on the surface of fly ash particles at different conditioning temperatures. There is still some CH on the surface, and most of the CH is encapsulated by major hydration products such as C-S-H gel and cannot be observed. With the increase of fly ash admixture, its pore structure was filled by hydration products. The hydration products, such as CH and C-S-H gel, lap each other and are tightly bound. The internal microstructure becomes stable, enhancing the densification of cementitious materials [31].

#### 3.2.3. Effect of Age of Maintenance on CH Content

**Figure 25:**
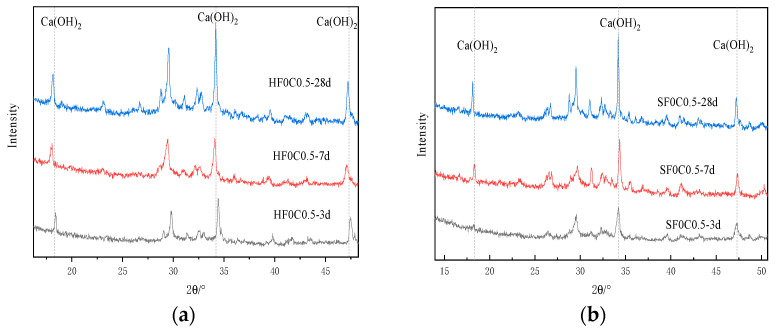
XRD plots of the effect of different age of curing on CH content. (**a**) 50 °C curing (**b**) Standard curing.

Figure 25 XRD shows plots of different ages under 50 °C curing and standard curing. It can be seen from the figure that the diffraction peak of CH increases with the increase in maintenance time. This indicates that the hydration reaction continues, and the generated CH content gradually increases.

The generation of CH has a similar trend, whether it is 50 °C curing or standard curing. In the 50 °C curing environment, the hydration reaction of cement is faster, so the production of CH is relatively higher. In the standard pile maintenance environment, the hydration reaction speed of cement is higher.

Although the generation of CH increases with increasing curing time, excessive CH content may reduce the strength and durability of concrete. Therefore, curing time and temperature must also be considered in concrete design and construction. Thus, the generation of CH can be controlled to obtain a concrete structure with good properties.

**Figure 26:**
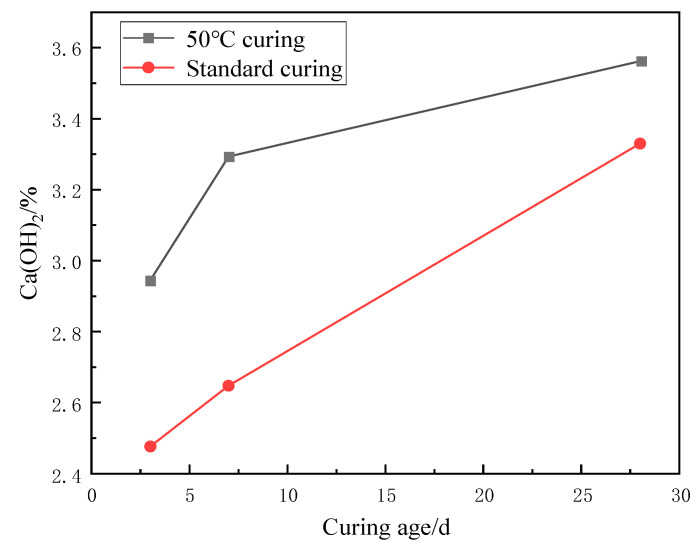
Effect of age of maintenance on CH content.

**Figure 27:**
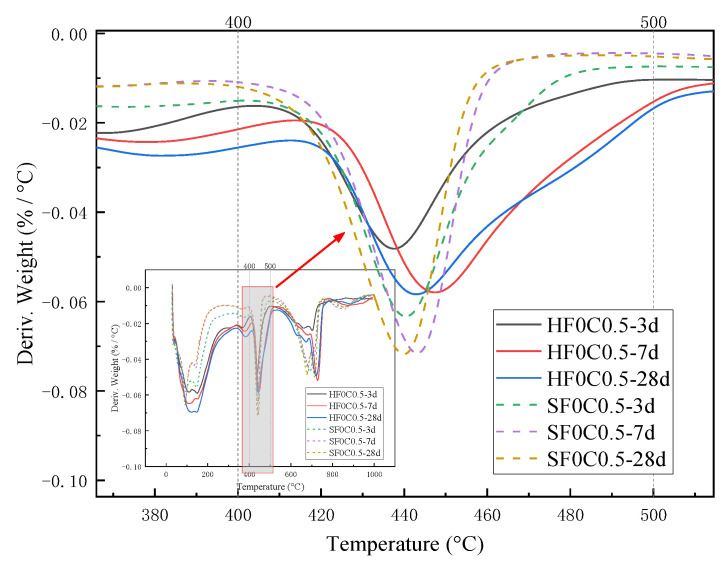
Effect of curing age on endothermic decomposition peak of CH.

Figure 26 and Figure 27 show the effect of age of curing on CH production. As the curing time increases, the hydration reaction of cement will continue, which also means that the production of CH will increase. For the same curing time, the CH content at 3, 7, and 28 days of curing decreased by 15.89%, 19.58%, and 6.51% at 50 °C curing compared to standard curing, respectively. The two main hydration reaction processes to generate CH are shown in Equations (8) and (9):

(8) $$2(3CaO\cdot SiO_{2})+6H_{2}O=3CaO\cdot 2SiO_{2}\cdot H_{2}O+3Ca{\left(OH\right)}_{2}$$

(9) $$2(2CaO\cdot SiO_{2})+4H_{2}O=3CaO\cdot 2SiO_{2}\cdot H_{2}O+Ca{\left(OH\right)}_{2}$$

Tricalcium silicate and dicalcium silicate react with water to form a C-S-H gel and CH. As the maintenance time increases, these reactions continue, and CH accumulates, increasing CH content.

**Figure 28:**
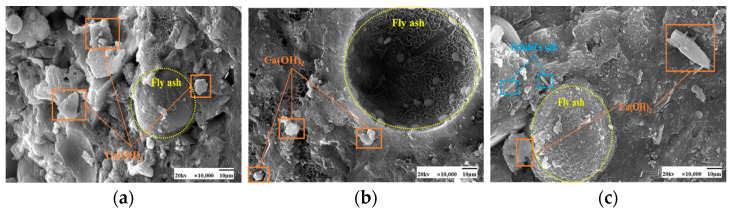
SEM images of cementitious materials hardened slurry at different curing ages in 50 °C curing. (**a**) 3 days (**b**) 7 days (**c**) 28 days.

**Figure 29:**
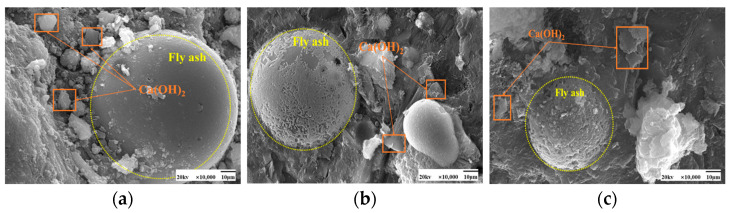
SEM images of cementitious materials hardened slurry at different curing ages in standard curing. (**a**) 3 days (**b**) 7 days (**c**) 28 days.

Figure 28 and Figure 29 show the microscopic morphology of hydration products at different curing ages under 50 °C curing and standard curing. It shows that the cement matrix of 50 °C curing is denser than standard curing, it is difficult to observe large pores, and the cement matrix contains a large amount of amorphous C-S-H gel. Under standard curing, CH crystals grew in the form of flakes. Under 50 °C curing, CH plate-like crystals are formed. With the prolongation of the maintenance time, the C-S-H gel and CH content increases.

### 3.3. Gray Correlation Analysis

Gray correlation analysis, as a rigorous method based on mathematical statistics, is dedicated to exploring and quantifying the relative correlation between different time series [38]. The core of this analytical framework lies in the use of the gray correlation coefficient to measure the closeness of two data series. Higher values of the correlation coefficient indicate more significant synergies between the series, while lower values of the correlation coefficient imply the presence of weaker interactions or correlations between the series.

One of the major strengths of the gray correlation model is its tolerance to the distributional characteristics of the data, i.e., it does not force the data to follow any particular pattern of probability distribution [38]. This feature enables gray correlation analysis to effectively reveal potential inter-sequence relationships in the face of limited sample size of data sets or ambiguous data distribution characteristics. In addition, the model is directly based on the intrinsic structure of sequence data for correlation measures. Therefore, it can significantly reduce the intervention of subjective judgment in the analysis process, thus enhancing the objectivity and reliability of the analysis results.

#### 3.3.1. Model Analysis

Suppose a set of data is divided into a number of sequences and a standard sequence is chosen as the reference sequence (denoted by $X_{0}\left(k\right)=\left\{X_{0}\left(1\right),X_{0}\left(2\right),X_{0}\left(3\right)\cdots X_{0}\left(n\right)\right\}$), other sequences as comparison sequences (denoted by $X_{i}\left(k\right)=\left\{X_{i}\left(1\right),X_{i}\left(2\right),X_{i}\left(3\right)\cdots X_{i}\left(n\right)\right\}$).

In view of the fact that the sequences are different in terms of magnitude and the magnitude of the values shows a significant disparity, the homogenization method is chosen for this experiment to make the original matrix dimensionless.

For each node k, the absolute difference between the comparison sequence *X_i_* and the reference sequence *X*_0_ is calculated:

(10) $${∆}_{i}\left(k\right)=\left|X_{0}\left(k\right)-X_{i}\left(k\right)\right|$$

Find the maximum value M and the minimum value m of all absolute differences.

Calculate the correlation coefficient $\varepsilon_{i}\left(k\right)$, Equation:

(11) $$\varepsilon_{i}\left(k\right)=\frac{m+\rho M}{{∆}_{i}\left(k\right)+\rho M}$$

where, ρ is the discrimination coefficient and is taken as 0.5.

Calculate the gray correlation:

(12) $$\gamma_{i}=\frac{1}{n}\sum_{k=1}^{n}\varepsilon_{i}\left(k\right)$$

#### 3.3.2. Discussion of Test Results

In this study, gray correlation analysis was used to quantitatively assess the relationship between the production of key hydration products during the hydration of cementitious materials and their influencing factors. By calculating the correlation coefficients under different conditions, the relevant data in Table 3, Table 4, Table 5 and Table 6 were obtained.

**Figure 30:**
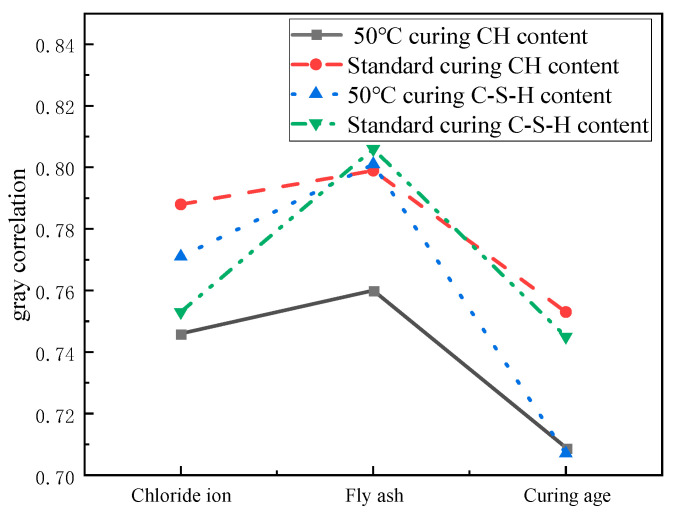
Gray correlation values of hydration products and influencing factors.

The range of gray correlation values is defined between 0 and 1. The increase of this value intuitively reflects the closeness of the link between the examined variable and the reference sequence (hydration product generation), which in turn indicates the importance of the variable [39]. Figure 30 represents the gray correlation values of hydration products with different influencing factors. The results of the study showed that the correlation between the content of C-S-H gel and CH and the fly ash admixture exhibited the most significant correlation under different curing temperature conditions. In particular, the gray correlation between the amount of C-S-H gel generation and the amount of fly ash admixture reached 0.801 under the 50 °C curing environment, while this value slightly increased to 0.806 under the standard curing condition. For CH generation, the correlation between 50 °C curing and fly ash admixture was 0.76, while the correlation under standard curing conditions was 0.799. This indicates that the effect of fly ash dosage on C-S-H gel and CH generation showed a strong correlation at both curing temperatures.

In addition, it was found that the correlation between the chloride dosage and the age of curing on CH generation under standard curing conditions reached 0.788 and 0.753, respectively, which were the highest among all the factors examined. This finding emphasizes the significant influence of chloride dosage and age on the CH generation process under specific curing conditions, further revealing its importance in the hydration mechanism of cement.

## 4. Conclusions

In this study, the mechanisms of the effects of chloride ions, fly ash, and curing conditions on the C-S-H gel and CH content of key components in cement hydration products were thoroughly investigated. The understanding of cement hydration products under different curing conditions was enriched. Meanwhile, the gray correlation of C-S-H gel and CH content with chloride ion, fly ash, and age of curing was clarified. However, there are other factors affecting the actual engineering which we will examine comprehensively in future research [40,41]. The specific conclusions are as follows:

(1) Chloride ions incorporated into the cement paste increased the concentration of calcium compounds with the increase in the dosage of chloride ions, thus increasing the amount of C-S-H gel production. At 1.3% chloride ion dosage, the content of C-S-H gel increased by 16.85% under 50 °C curing compared with the control group. It increased by 14.2% under standard curing compared with the control group.
(2) Fly ash is rich in active SiO_2_ and AI_2_O_3_, which can react with CH generated by cement hydration to produce C-S-H gel. Compared with standard curing, 50 °C curing can stimulate the activity of fly ash and generate a large amount of C-S-H gel. The content of C-S-H gel increased by 13.55% under 50 °C curing compared with the control group when 25% of fly ash was added. The CH content decreased by 34.18% under 50 °C curing conditioning compared to control.
(3) The microstructure of cement paste under 50 °C curing accumulates the hydration products with the increase of curing age, and the microstructure formed is more compact compared with that of standard curing. The contents of C-S-H gel and CH increased most at the elevated curing temperature at 7 days of curing, increasing by 9.35% and 19.58%, respectively.
(4) The content of C-S-H gel and CH had the greatest gray correlation with fly ash dosage. The correlation between the content of C-S-H gel and the fly ash dosage was 0.801 and 0.806 at 50 °C curing and standard curing, respectively. The correlation between CH content and fly ash dosage was 0.76 and 0.799 at 50 °C curing and standard curing, respectively. The highest correlation between the chloride dosage and age of maintenance and the CH content under standard curing were 0.788 and 0.753, respectively.

## Figures and Tables

**Table 1:** Chemical composition.

Chemical Composition	SiO_2_	CaO	Al_2_O_3_	Fe_2_O_3_	MgO	K_2_O	Na_2_O	SO_3_
Content in fly ash /%	53.80	3.16	24.60	9.32	1.52	0.82	0.28	0
Content in cement /%	21.90	59.31	6.26	3.79	1.63	0	0	2.41

**Table 2:** Cement net mortar mixing ratio.

Specimen Number	Water/g	Cement/g	Fly Ash/g	Water Reducing Agent/g	Cl^−^ Content/%	Maintenance Environment
F0C0	200	500	0	1	0	20 °C curing (Standard curing) 50 °C curing (High temperature curing)
F15C0	200	425	75	1	0	
F25C0	200	375	125	1	0	
F0C0	200	500	0	1	0.5	
F15C0.5	200	425	75	1	0.5	
F25C0.5	200	375	125	1	0.5	
F0C0.5	200	500	0	1	1.3	
F15C1.3	200	425	75	1	1.3	
F25C1.3	200	375	125	1	1.3	

Note: in the specimen number “H” for 50 °C curing, “S” for standard curing, and “F” for fly ash, the value behind the letter represents the admixture of fly ash accounted for the mass percentage of cementitious materials. “F” means fly ash, and the value after the letter represents the mass percentage of fly ash in the cementitious material. “d” denotes the maintenance time. “C” denotes chloride ions, and the value after the letter represents the mass percentage of chloride ions in water.

**Table 3:** Correlation coefficients between CH production and each factor under 50 °C curing.

CH Content	Chloride Ion	Fly Ash	Curing Age
2.412	0.488	0.831	0.699
2.308	0.889	0.800	0.677
2.361	0.339	0.816	0.688
3.462	0.739	0.398	1.000
2.506	0.964	0.861	0.719
2.361	0.908	0.481	0.688
2.945	0.889	1.000	0.472
3.293	0.782	0.867	0.486
3.562	0.716	0.786	0.958

**Table 4:** Correlation coefficients between CH production and various factors under standard curing.

CH Content	Chloride Ion	Fly Ash	Curing Age
2.822	0.438	0.937	0.761
2.747	1.000	0.908	0.742
2.775	0.339	0.918	0.749
3.330	0.788	0.397	0.926
3.127	0.851	0.961	0.853
2.775	0.987	0.497	0.749
2.477	0.916	0.816	0.513
2.648	0.987	0.872	0.557
3.330	0.788	0.882	0.926

**Table 5:** Correlation coefficients between C-S-H production and various factors under 50 °C curing.

C-S-H Content	Chloride Ion	Fly Ash	Curing Age
1.220	0.406	0.860	0.689
1.327	0.890	0.967	0.756
1.513	0.333	0.842	0.908
1.22	0.995	0.406	0.689
1.356	0.864	1.000	0.776
1.377	0.845	0.486	0.791
1.064	0.839	0.741	0.483
1.297	0.920	0.934	0.482
1.377	0.845	0.975	0.791

**Table 6:** Correlation coefficients between C-S-H production and various factors under standard curing.

C-S-H Content	Chloride Ion	Fly Ash	Curing Age
1.358	0.410	0.931	0.743
1.403	0.903	0.975	0.771
1.502	0.336	0.940	0.839
1.403	0.903	0.402	0.771
1.517	0.814	0.926	0.850
1.542	0.797	0.528	0.870
0.961	0.742	0.669	0.549
1.305	0.997	0.885	0.518
1.442	0.871	1.000	0.796

## Data Availability

The original contributions presented in the study are included in the article, further inquiries can be directed to the corresponding author.
